# Smartphone and Tablet Usage during COVID-19 Pandemic Confinement in Children under 48 Months in Barcelona (Spain)

**DOI:** 10.3390/healthcare9010096

**Published:** 2021-01-19

**Authors:** Àurea Cartanyà-Hueso, Cristina Lidón-Moyano, Pia Cassanello, Ana Díez-Izquierdo, Juan Carlos Martín-Sánchez, Albert Balaguer, Jose M. Martínez-Sánchez

**Affiliations:** 1 Group of Evaluation of Health Determinants and Health Policies, Department of Basic Sciences, Universitat Internacional de Catalunya, 08195 Sant Cugat del Vallès, Spain; acartanya@uic.es (À.C.-H.); clidon@uic.es (C.L.-M.); adiez@uic.es (A.D.-I.); jcmartin@uic.es (J.C.M.-S.); 2 Pediatric Division, Hospital Universitari General de Catalunya, 08159 Sant Cugat del Vallès, Spain; mariapiacassanello@gmail.com (P.C.); abalaguer@uic.es (A.B.); 3 Department of Medicine, Universitat Internacional de Catalunya, 08159 Sant Cugat del Vallès, Spain; 4 Pediatric Allergy and Pulmonology Section, Department of Pediatrics, Hospital Universitari Vall d’Hebron, 08035 Barcelona, Spain

**Keywords:** coronavirus infections, pandemics, child, preschool, infant, screen time, Spain

## Abstract

Background: Total lockdown due to COVID-19 pandemic might have potentially increased screen time in children. This study aims to describe the smartphone and tablets usage in children under 48 months living in Barcelona during the COVID-19 confinement. Methods: Cross-sectional study using a non-probabilistic sample of parents with children under 48 months living in Barcelona (Spain) during COVID-19 confinement (n = 313). We calculated percentages of exposure to smartphones and tablets. Moreover, for those children were exposed, we calculated unadjusted and adjusted Geometric Mean Ratios (GMR) of daily smartphones and tablets usage and their 95% confidence intervals (95% CI) trough Generalized Linear Models with Gamma family and link log. Associations were adjusted for potential confounders. Results: During COVID-19 confinement, 67.5% of children under 48 months were daily exposed to smartphones and tablets. Further, those children who were exposed during meals, as well as before going to bed, spend longer durations using them, aGMR = 2.38 (95% CI 1.73, 3.34) and aGMR = 1.95 (95% CI 1.34, 2.91) respectively. Conclusion: Two out of three children under 48 months living in Barcelona were daily exposed to smartphones and tablets during total lockdown due to COVID-19. Taking this findings into account cohort studies are needed to assess any change in the screen time patterns due to total confinement in order to allow the Government help families, particularly those more vulnerable, in a possible pandemic resurgence.

## 1. Introduction

Since SARS-CoV-2 (Severe Acute Respiratory Syndrome Coronarivus 2) came up into our lives in December 2019 Governments applied measures that directly affected children, such as 1.57 billion children were unable to go to school [1]. Spain was one of the countries that applied hardest restrictions in order to avoid COVID-19 transmission, making more than 2 million of children under 5 years totally confined around 45 days, since the first measure applied by Government was closing kindergartens, schools, and high-schools and banning children and teenagers going out [2].

Totally locked due to SARS-CoV-2 pandemic might have altered children routines. Firstly, children might have increased high-calorie food consumption, in this sense a previous study show that during COVID-19 confinement people changed their dietary patterns as a way of facing the anxiety and boredom up, and because they did not feel motivated enough to follow a healthy diet [3]. Regarding to Spain, in addition to previous reasons, our supposition is this change in the eating behaviour also might be due to parents had to work from home, at the same time, had to take care of their children, making that some parents might have had less time for cooking, and consequently, they might have recurred to precooked food, fast food, or poor-nutrient products. Further, a decline of physical activity, due to children were banned to go out, and besides, some homes were not big enough and/or had not outdoors areas, such as balconies, terraces, or gardens, making children were sitting most of the time. In this sense, during COVID-19 confinement children behaved more sedentary including an increment in the daily screen time since might have been a usual way to entertain children while parents have worked from home, and besides, video calls were the predominant via of communication that children had with other family members and friends.

During the last decade the screen time usage in children constantly change, having the first contact sooner and increasing the variety and the accessibility of screen devices and media content [4]. Wide evidence suggests excessive screen time in childhood and adolescence is related to great variety of physiological and psychological issues [5], as well as for the youngest [6]. Hence, firstly American Academy of Pediatarics (AAP) in 2016 and more recently World Health Organization (WHO) in 2019 recommended that those children below 2 years should not be exposed to screens and those from 2 years to 5 years the daily screen time should be limited to 1 h [7,8]. However, the percentages of exceeding 1 h of screen time in 2017 were 40.9% and 66.8% for those children from 1 and 2 years and from 3 to 5 years respectively [9].

Due to the exceptionality of the moment and emergent knowledge of the possible adversary effects of excessive screen time on children’s health, the aim of this study is to describe the smartphone and tablet usage, including if they use during meals and before going to bed, in children under 48 months living in Barcelona during the COVID-19 total lockdown.

## 2. Methods

Cross-sectional study using a sample of parents with children under 48 months living in Barcelona (Spain) (n = 313) included in the EpiSon-II study, which is the second phase of EpiSon study. The aim of EpiSon-II study is to study the sleep quality of children during the confinement due to COVID-19. More detailed information about EpiSon study can be found elsewhere [10]. The sampling of EpiSon-II study was non-probabilistic and the sample was gathered through online questionnaires (www.epison.es). Paediatricians of Hospital Universitari General de Catalunya (HUGC) spread the information of the study and the questionnaire within parents of patients, and besides, paediatricians encouraged parents to spread the questionnaire within their contacts and through online free-access parental support groups. To gain access to completing the survey, participants filled in the informed consent online. Previously, we got the approval from the Ethics Committee of the HUGC and the Ethics Committee of Research (CER) of the Universitat Internacional de Catalunya (UIC-Barcelona). The sample was recruited from April to June 2020. Exclusion criteria were (1) not to live in Barcelona (n = 8) and (2) to have missing data in smartphone and tablet usage during confinement variables (n = 3). Therefore, the final sample included 302 parents with children under 48 months living in Barcelona. Assuming an expected prevalence of being exposed of 50%, an alpha error of 5% and precision of 6% the theoretical sample size is 267 individuals, therefore our sample achieve enough statistical power.

### 2.1. Daily Smartphone and Tablet Usage during Confinement

Daily smartphone and tablet usage during confinement variable was obtained through the question “During the confinement, how much time, in minutes, on average did your child use or did you show to your child a smartphone or tablet?”. We dichotomized to be daily exposed to smartphones and tablets during confinement (1) no: those children reported 0 minutes and (2) yes: those children reported more than 0 minutes. Further, those parents who answered that their children were exposed, reported if during COVID-19 confinement their children were exposed to smartphones and tablets during meals (yes/no) and before going to bed (yes/no).

### 2.2. Potential Confounders and Covariates

Based on the literature we selected the following variables as potential confounders [11,12,13,14]. Features of children: age in months (0–11, 12–23, 24–35, and 36–48), since evidence shows that older children spent longer duration of screen time [11]. Further, features of father or mother, depending who answered the questionnaire: relationship between him/her and children (mother/father), since evidence shows that maternal variables had more effect on behaviour of the children than paternal variables [11], education level (high: university education; medium: high school and training cycles; low: unschooled, elementary school completed or uncompleted and special education), since evidence shows that children belonged families with low socio-education level were more likely to be exposed longer periods of screen time [12,13,14], and age in years (18–34, 35–39 and ≥40), since previous researches find weak association between maternal age and screen time usage [11]. Finally, we used as potential confounder if parents answered questionnaire after 26 April 2020 [15], which is the day that government relaxed COVID-19 confinement restrictions and allowed children going out 1 h daily (no/yes), although the questions were based on total confinement, parents who answered the questionnaire after Government relaxed the restrictions were more susceptible to recall bias.

Additionally, we used as covariates sex of the children (male/female), if children had siblings (no/yes), and if children went to a kindergarten (no/yes), the last covariate included only those children were born after 1 January 2017.

### 2.3. Statistical Analysis

We calculated the percentages of not being and being exposed to smartphones and tablets during COVID-19 confinement overall and stratified by potential confounding variables. Further, for those children were exposed to them during COVID-19 confinement we calculated median and interquartile range (IQR: first and third quartile) of daily smartphone and tablet usage during COVID-19 confinement, due to the not normal distribution of the variable. Further, we calculated the percentage of being exposed to smartphones and tablets during COVID-19 confinement meals and before going to sleep during the COVID-19 confinement. Additionally, we calculated unadjusted and adjusted prevalence ratios (PR) with their 95% confidence intervals (95% CI) of being exposed to smartphones and tablets during COVID-19 confinement according to potential confounding variables. PR were fitted through Generalized Linear Models (GLM) with Poisson family and robust variances [16]. Finally, for those children were exposed to smartphones and tablets during COVID-19 confinement, we calculated the unadjusted and adjusted geometric mean ratios with their 95% CI of daily smartphones and tablets usage during COVID-19 confinement average trough GLM with Gamma family and link log [17]. Associations were adjusted for potential confounding variables. The statistical program used was R-3.5.2.

## 3. Results

### 3.1. Descriptive of the Sample

This study included 302 children under 48 months, 50.3% were female, the median of the age were 22 months (IQR: 11–33.75), 62.9% had siblings, 96.0% questionnaires were answered by mothers, 78.1% of parents had high education level, and 42.2% of parents were between 35 and 39 years.

### 3.2. Smartphone and Tablet Usage during Total COVID-19 Lockdown

67.5% of children under 48 months were daily exposed to smartphones and tablets during COVID-19 confinement. The percentage of being exposed to smartphones and tablets during confinement increased according to the age of the child, rising from 38.5% to 87.1%, as well as, the percentage of being exposed to smartphones and tablets during COVID-19 confinement meals, ranging from 16.7% to 37.0%, and of being exposed to smartphones and tablets before going to bed during COVID-19 confinement ranging from 10.0% to 27.8%. Regarding to educational level, those children whose parents had lower education level were more likely to be exposed to smartphones and tablets during COVID-19 confinement meals (Table 1).

Table 2 shows that daily smartphone and tablet usage during confinement were higher for those children were exposed to them during COVID-19 confinement meals and before going to bed during COVID-19 confinement, these associations were maintained once we adjusted for potential confounders (Table 2).

## 4. Discussion

During COVID-19 confinement, around two out of three children and one out of two children under 48 months and under 24 months living in Barcelona (Spain) were exposed to smartphones and tablets, respectively. Moreover, when children were totally locked due to the SARS-CoV-2 pandemic, those children who were exposed to screen during meals, as well as, before going to bed, spend longer periods in front of smartphones and tablets.

Children were exposed to smartphones and tablets in meals during COVID-19 presented longer periods using smartphones and tablets. Our results are consistent with those obtained for a Lithuanian study published in 2019 which showed the prevalence and the associated factors of screen use during meals in children aged from 2 to 5 years [18]. Further, previous literature showed that exposing children to screens during meals in the early childhood is linked to worse dietary patterns [19]. To be exposed to screens during meals might have an effect on the development of autonomous eating habits and self-regulatory skills [18]. Moreover, our findings suggest that those children were exposed to smartphones and tablets before going to bed had higher patterns of daily smartphones and tablets usage. Using screen devices just before going to bed gets worse sleep, since the light from the screens stimulates brain activity and supress melatonin production [20].

Our results show that about three out of four of children from 12 months to 47 months were exposed to smartphones and tablets during COVID-19 confinement. This result is slightly lower than those obtained in the 2017 National Health Survey (NHS) (82.3% in 2017 NHS vs. 77.7% in our study) [21]. This discrepancy might be due to (1) in our study there was an overrepresentation of those children whose parents had higher education level [22], as previous evidence suggests those children belonged to lower education family were more likely to be exposed to screens [12,13,14], (2) 2017 NHS included in this percentage those children who were never exposed to screens, as well as, those children who were almost never exposed to screens, and (3) our study only included smartphone and tablet exposure.

Our results agreed with previous literature showing positive association between time spent in front of smartphones and tablets and age of the children [11]. In reference to educational level our findings do not suggest negative association between being exposed to smartphones and tablets and education level of the parents, in contrast to evidence [12,13,14]. This discrepancy might be due to parents with high education level were overrepresented [22]. Considering only those children were exposed, those children had lower educated parents spent longer periods in front of smartphones and tablets, as well as, were more likely to be exposed during meals.

Additionally, we do not find association between to be exposed to smartphones and tablets during COVID-19 confinement and age of the mother/father, nor between daily smartphones and tablets udage during COVID-19 confinement and age of the mother/father. Regarding that Duch et al. [11] concluded an unclear association between maternal age and screen time, our results might provide more evidence that there is not association between age of mother/father and screen time in children.

Nowadays, screen time patterns in children are based on smartphone and tablets exposure, unlike few years ago where the screen time patterns were based on TV exposure. In this sense, taking into account the existed research gap of studies including these devices, our work provides more evidence of the updated screen time patterns in children. However, more evidence is needed of the screen time patterns of these screen devices during COVID-19 pandemic, as well as, before and after COVID-19 pandemic.

Children, as well as other vulnerable collectives, were those that spend most time confined. First measures applied by Government were to close kindergartens, schools, and high-schools and to ban children of going out [2]. These restrictions might have had an effect on children’s health, such as schools closures might have made that during COVID-19 confinement might have eaten less healthily, since schools are not only a place for learning, but also a place for guaranteeing that some children eat healthily at least once [23]. In addition, the effect that might have had this quarantine on psychological well-being of the children is already unclear. In this context, a recent systematic review showed that children presented better prognostic and softer cases than adults [24], and besides, children do not seem to be the main transmitters [25].

### Strengths and Limitations

This study is the first study to describe smartphones and tablets usage in children during COVID-19 lockdown. Moreover, this study focuses on smartphone and tablets exposure in toddlers and pre-schoolers adding more evidence of current screen time patterns. Nevertheless, this study also has some limitations. Firstly, final sample was gathered through non-probabilistic sampling, therefore, final sample might not be representative of Barcelona. We have compared our sample with 2019 municipal population register of Barcelona and we found certain limitations, girls and children whose parents had high education level are overrepresented and children were under 12 months and between 36 and 47 months are underrepresented [22,26]. Moreover, this study does not consider some important information that might be related to daily smartphones and tablets usage during COVID-19 confinement, such as if the parents had to work from home, size of home, if children lived in homes with outdoors spaces, in this sense we assume that families with higher education level were more likely to have bigger homes and homes with outdoor areas, or if children use screen devices to connect with school. Additionally, due to the cross-sectional design of the study, we can not assess causality and temporal sequence between COVID-19 confinement and screen time. Finally, on account of data was obtained through online questionnaire we cannot discard some sources of bias, with most concern in the selection bias [27].

## 5. Conclusions

Two out of three children under 48 months living in Barcelona were exposed to smartphones and tablets during confinement due to COVID-19 pandemic. Considering our findings and evidence shows that excessive screen time may have adverse effects on children’s health. Cohort studies are needed to confirm any change in the screen time patterns due to COVID-19 total confinement in order to allow the Government help families, particularly those more vulnerable, in a possible pandemic resurgence.

## Figures and Tables

**Table 1:** Percentage of children exposed to smartphones and tablets, median and interquartile range of daily smartphone and tablets usage, and percentages of children exposed to smartphones and tablets during meals and before going to bed during COVID-19 confinement overall and according to potential confounders.

		To Be Exposed to Smartphones or Tablets during COVID-19 Confinement		Daily Smartphone and Tablet Usage during Confinement (min) ^4^		To Be Exposed to Smartphones and Tablets during COVID-19 Confinement Meals ^4^		To Be Exposed to Smartphones and Tablets before Going to Bed during COVID-19 Confinement ^4^	
	n (%)	n (%)	*p*-Value	Median (IQR)	*p*-Value	n (%)	*p*-Value	n (%)	*p*-Value
**Overall**	302 (100)	204 (67.5)		45 (15–90)		58 (28.6)		41 (20.2)	
**Sex of the child**			0.839 ^5^		0.895 ^8^		0.002 ^5^		0.222 ^5^
Male	150 (49.7)	104 (68.4)		60 (15–60)		40 (38.5)		25 (24.0)	
Female	152 (50.3)	100 (66.7)		31.5 (15–90)		18 (18.2)		16 (16.2)	
**Age of the child (months)**			<0.001 ^6^		<0.001 ^9^		0.011 ^6^		0.011 ^6^
0–11	78 (25.8)	30 (38.5)		22.5 (6.75–60)		5 (16.7)		3 (10.0)	
12–23	85 (28.1)	53 (62.4)		30 (15–60)		10 (19.2)		6 (11.5)	
24–35	77 (25.5)	67 (87.0)		60 (30–120)		23 (34.3)		17 (25.4)	
36–47	62 (20.5)	54 (87.1)		60 (30–90)		20 (37.0)		15 (27.8)	
**Siblings**			0.968 ^5^		0.508 ^8^		0.240 ^5^		0.599 ^5^
No	112 (37.1)	75 (67.0)		45 (15–67.5)		17 (23.0)		13 (17.6)	
Yes	190 (62.9)	129 (67.9)		45 (15–90)		41 (31.8)		28 (21.7)	
**Relationship between who answered the questionnaire and child**			0.349 ^7^		0.726 ^8^		1.000 ^7^		0.218 ^7^
Mother	290 (96)	194 (66.9)		45 (15–90)		55 (28.5)		41 (21.2)	
Father	12 (4)	10 (83.3)		33 (30–60)		3 (30.0)		0 (0.0)	
**Education level of mother/father ^1^**			0.525 ^6^		0.041 ^9^		0.022 ^6^		0.786 ^6^
High	235 (78.1)	162 (68.9)		30 (15–60)		41 (25.5)		33 (20.5)	
Medium	46 (15.3)	27 (58.7)		60 (30–90)		8 (29.6)		4 (14.8)	
Low	20 (6.6)	14 (70.0)		60 (30–108.75)		8 (57.1)		3 (21.4)	
**Age of mother/father (years)**			0.496 ^6^		0.255 ^9^		0.915 ^6^		0.249 ^6^
18–34	106 (38.3)	68 (64.2)		30 (15–60)		18 (26.5)		17 (25.0)	
35–39	117 (42.2)	81 (69.2)		36 (15–90)		22 (27.2)		14 (17.3)	
≥40	54 (19.5)	37 (68.5)		60 (30–90)		9 (24.3)		7 (18.9)	
**Kindergarten ^2^**			<0.001 ^5^		0.027 ^8^		0.627 ^5^		0.283 ^5^
No	117 (43.2)	59 (50.4)		30 (7.5–60)		14 (23.7)		8 (13.6)	
Yes	154 (56.8)	117 (76.0)		45 (24–75)		33 (28.9)		25 (21.9)	
**Questionnaire was answered after government allowed children going out 1 h/day ^3^**			1.000 ^5^		<0.001 ^8^		<0.001 ^7^		<0.001 ^7^
No	20 (6.6)	13 (65.0)		30 (30–60)		3 (23.1)		2 (15.4)	
Yes	282 (93.4)	191 (67.7)		45 (15–90)		55 (28.9)		39 (20.5)	

IQR: interquartile range, first and third quartile. ${}^{1}$ High: university education; medium: high school and training cycles; low: unschooled, elementary school completed or uncompleted and special education. ${}^{2}$ Includes those children were born after 01/01/2017. ${}^{3}$ If parents answered questionnaire after 26 April 2020, which is the day that government relaxed COVID-19 confinement restrictions and allowed children going out 1 h/day. ${}^{4}$ Includes those children were exposed to smartphones and tablets during COVID-19 confinement (n = 204). ${}^{5}$*p*-value obtained through Chi-square test. ${}^{6}$*p*-value obtained through Chi-square trend test. ${}^{7}$*p*-value obtained through Fisher exact test. ${}^{8}$*p*-value obtained through Mann-Whitney test. ${}^{9}$ *p*-value obtained through Kruskal-Wallis test.

**Table 2:** Prevalence ratio (unadjusted and adjusted with their 95% confidence intervals) of being exposed to smartphones and tablets during COVID-19 confinement according to potential confounders, and geometric mean ratios (unadjusted and adjusted with their 95% confidence intervals) of daily smartphone and tablet usage during COVID-19 confinement according to potential confounders.

	To Be Exposed to Smartphones and Tablets during COVID-19 Confinement		Daily Smartphone and Tablets Usage during COVID-19 Confinement ${}^{1}$	
	PR (95% CI)	aPR ${}^{4}$ (95% CI)	GMR (95% CI)	aGMR ${}^{4}$ (95% CI)
**To be exposed to screens during** ** COVID-19 confinement meals**				
No	———	———	1.00 Reference	1.00 Reference
Yes	———	———	2.42 (1.82, 3.25)	2.38 (1.73, 3.34)
**To be exposed to screens before going to** ** bed during COVID-19 confinement**				
No	———	———	1.00 Reference	1.00 Reference
Yes	———	———	2.00 (1.41, 2.91)	1.95 (1.34, 2.91)
**Sex of the child**				
Male	1.00 Reference	1.00 Reference	1.00 Reference	1.00 Reference
Female	0.97 (0.83, 1.14)	0.96 (0.83, 1.12)	1.00 (0.73, 1.36)	0.99 (0.71, 1.38)
**Age of the child (months)**				
0–11	1.00 Reference	1.00 Reference	1.00 Reference	1.00 Reference
12–23	1.62 (1.17, 2.25)	1.42 (1.01, 1.99)	1.19 (0.72, 1.91)	1.31 (0.76, 2.21)
24–35	2.26 (1.69, 3.03)	2.16 (1.61, 2.89)	2.07 (1.28, 3.27)	2.10 (1.23, 3.52)
36–47	2.26 (1.68, 3.05)	2.15 (1.59, 2.90)	1.88 (1.14, 3.02)	2.11 (1.22, 3.59)
**Siblings**				
No	1.00 Reference	1.00 Reference	1.00 Reference	1.00 Reference
Yes	1.01 (0.86, 1.19)	0.92 (0.79, 1.09)	1.04 (0.75, 1.43)	0.95 (0.67, 1.34)
**Relationship between who answered** ** the questionnaire and child**				
Mother	1.00 Reference	1.00 Reference	1.00 Reference	1.00 Reference
Father	1.25 (0.96, 1.62)	1.17 (0.87, 1.57)	0.91 (0.48, 2.03)	1.11 (0.51, 2.73)
**Education level of mother/father ${}^{2}$**				
High	1.00 Reference	1.00 Reference	1.00 Reference	1.00 Reference
Medium	0.85 (0.66, 1.10)	0.85 (0.67, 1.08)	1.28 (0.84, 2.05)	1.36 (0.84, 2.30)
Low	1.02 (0.75, 1.37)	0.93 (0.67, 1.28)	1.66 (0.96, 3.19)	1.47 (0.76, 3.23)
**Age of mother/father (years)**				
18–34	1.00 Reference	1.00 Reference	1.00 Reference	1.00 Reference
35–39	1.08 (0.90, 1.30)	1.01 (0.85, 1.20)	1.10 (0.75, 1.59)	0.97 (0.66, 1.40)
≥40	1.07 (0.85, 1.34)	0.91 (0.73, 1.12)	1.20 (0.76, 1.93)	0.99 (0.62, 1.60)
**Questionnaire was answered** ** after government allowed children** **going out 1 h/day** ${}^{3}$				
No	1.00 Reference	1.00 Reference	1.00 Reference	1.00 Reference
Yes	1.04 (0.75, 1.45)	1.04 (0.72, 1.50)	1.57 (0.79, 2.78)	1.94 (0.93, 3.59)

PR: prevalence ratio; aPR: adjusted prevalence ratio; 95% CI: 95% confidence interval GMR: geometric mean ratio obtained through generalized linear model with Gamma family and link log aGMR: adjusted geometric mean ratio obtained through generalized linear model with Gamma family and link log. ${}^{1}$ Includes those children were exposed to smartphones and tablets during COVID-19 confinement (n = 204). ${}^{2}$ High: university education; medium: high school and training cycles; low: unschooled, elementary school completed or uncompleted and special education. ${}^{3}$ If parents answered questionnaire after 26 April 2020, which is the day that government relaxed COVID-19 confinement restrictions and allowed children going out 1 h/day. ${}^{4}$ Adjusted for age of the children, relationship between who answered the questionnaire and child, education level of mother/father, age of mother/father, and if parents answered questionnaire after government allowed children going out 1 h/day.
